# Nanomechanical Properties of Rib Bones in Diabetic vs. Healthy Rat Models

**DOI:** 10.3390/nano15201582

**Published:** 2025-10-17

**Authors:** Tamás Tarjányi, Csaba Rosztóczy, Ferenc Peták, Fruzsina Kun-Szabó, Gábor Gulyás, József Tolnai, Krisztián Bali, Petra Somogyi, Rebeka Anna Kiss, Gergely H. Fodor

**Affiliations:** 1 Department of Medical Physics and Informatics, University of Szeged, 6720 Szeged, Hungary; rosztoczycsaba@gmail.com (C.R.); petak.ferenc@med.u-szeged.hu (F.P.); kun-szabo.fruzsina.anna@med.u-szeged.hu (F.K.-S.); tolnai.jozsef@med.u-szeged.hu (J.T.); somogyi.petra@med.u-szeged.hu (P.S.); kissrebeka0125@gmail.com (R.A.K.); fodor.gergely@med.u-szeged.hu (G.H.F.); 2 Department of Optics and Quantum Electronics, University of Szeged, 6720 Szeged, Hungary; gulyasg@titan.physx.u-szeged.hu; 3 SEMILAB Semiconductor Physics Laboratory Co., Ltd., 1117 Budapest, Hungary; krisztian.bali@semilab.hu; 4 Department of Cell Biology and Molecular Medicine, University of Szeged, 6720 Szeged, Hungary

**Keywords:** nanoindentation, diabetes mellitus, rib bone, dynamic loading, aging

## Abstract

This study examines how diabetes mellitus and physiological aging influence the nanomechanical behavior of rat rib cortical bone using combined static and dynamic nanoindentation. Ribs from young control, old, and streptozotocin-induced diabetic rats were analyzed to quantify both intrinsic and frequency-dependent mechanical properties. Static nanoindentation revealed markedly higher hardness and elastic modulus in the diabetic group (0.47 ± 0.22 GPa and 9.53 ± 3.03 GPa, respectively) compared to controls (0.11 ± 0.03 GPa and 3.21 ± 0.51 GPa; *p* < 0.001). The modulus-to-hardness ratio, an indicator of fracture toughness, was reduced from 30.34 in controls to 20.45 in diabetics, suggesting increased stiffness but greater brittleness. Dynamic nanoindentation (0–4.5 Hz) demonstrated significant aging-related changes in the storage and loss moduli (*p* < 0.001), while the loss factor (tan δ < 1) and viscosity remained similar across groups, indicating predominantly solid-like behavior. These results show that diabetes stiffens bone tissue through matrix-level alterations, whereas aging primarily affects its viscoelastic damping capacity. The combined static–dynamic nanoindentation protocol provides a robust framework for distinguishing disease- and age-related bone degradation at the tissue scale. Translationally, the findings help explain why bones in diabetic or elderly individuals may fracture despite normal mineral density, underscoring the need to assess bone quality beyond conventional densitometry.

## 1. Introduction

Mechanical performance of bone arises from its hierarchical composite architecture, in which mineralized collagen fibrils and their interfaces govern tissue-level stiffness and strength [1,2,3]. Nanoindentation enables localized, sub-micron probing of this mineral–collagen composite and has been widely used to quantify elastic modulus and hardness in cortical and trabecular bone under physiological and pathological conditions [4,5]. Foundational studies established benchmark tissue-level properties and demonstrated how lamellar and microstructural heterogeneity map to mechanical variation at the indentation scale [6].

Among systemic factors that impair bone quality, diabetes mellitus and physiological aging both increase skeletal fragility through distinct but overlapping mechanisms. Chronic hyperglycemia promotes the accumulation of advanced glycation end products (AGEs) that alter collagen cross-linking and mineral–matrix interactions, leading to reduced hardness and stiffness [7,8,9,10,11]. Aging, in turn, is characterized by collagen degradation, mineral heterogeneity, and loss of matrix-bound water, resulting in higher brittleness and diminished energy dissipation [12,13,14]. Most previous nanoindentation studies have concentrated on long bones such as the femur or tibia [12,15], providing valuable insight into cortical and trabecular mechanics under systemic conditions. In contrast, the rib remains underexplored despite its dual mechanical relevance: it not only contributes to thoracic stability but also experiences repetitive low-amplitude microstresses during tidal ventilation [16,17,18]. These cyclic deformations continuously test the viscoelastic damping capacity of the bone, making rib tissue a sensitive indicator of how aging and diabetes alter nanoscale resilience and energy dissipation under physiologically relevant loading.

Most prior research has relied on quasi-static indentation protocols that capture hardness and elastic modulus but overlook the time- and frequency-dependent viscoelastic response of bone. Dynamic nanoindentation allows simultaneous measurement of storage modulus (E′), loss modulus (E″), and loss factor (tan δ), providing a more comprehensive characterization of the mineral–collagen composite [19,20,21]. This frequency-resolved approach is particularly relevant for rib tissue, which undergoes continuous cyclic loading during tidal ventilation, thereby allowing laboratory measurements to reflect physiologically meaningful, dynamic microstresses. However, systematic comparisons integrating both static and dynamic parameters across healthy, old, and diabetic bones, particularly in ribs, remain limited.

In this study, we characterize the nanomechanical properties of rat rib bone under the following three conditions: young healthy, old healthy, and diabetic, by combining static nanoindentation for hardness and elastic modulus with a multifrequency dynamic protocol to obtain viscoelastic parameters. We hypothesized that diabetes primarily reduces static stiffness and hardness due to compromised collagen quality, whereas aging more strongly affects the frequency-dependent viscoelastic response. This combined approach provides a compact and reproducible method to assess frequency-dependent bone quality and differentiate disease- and age-related mechanical signatures at the tissue scale.

## 2. Materials and Methods

### 2.1. Animal Model and Sample Preparation

The left 8th ribs were harvested from Sprague–Dawley rats immediately post-euthanasia. Three groups of animals (*n* = 11) were used for the study as follows: a normal control group with a young age of 18 weeks (*n* = 4), an 18-week-old diabetic group including rats treated with streptozotocin (65 mg/kg) 12 weeks before the euthanasia [22] (*n* = 4), and a group with elderly rats including animals with an age of 2 years old (*n* = 3). Prior to sample collection, the animals were euthanized using sodium pentobarbital at a dose of 200 mg/kg body mass via intravenous injection. After the euthanasia, surgical removal of the lower ribs was performed under sterile conditions. The harvested rib bones were cleaned of the surrounding soft tissue, and they were placed in a freezer to store for 38 weeks at a temperature of −80 °C.

To excessively defrost, the bone samples were placed at 4 °C for 24 h. To dehydrate the samples, they went through a graded ethanol series to also preserve structural integrity in the following series: 70% and 80% ethanol for 24 h, 90% for 72 h, and 100% for 72 h, followed by xylene solution for 48 h.

**Figure 1:**
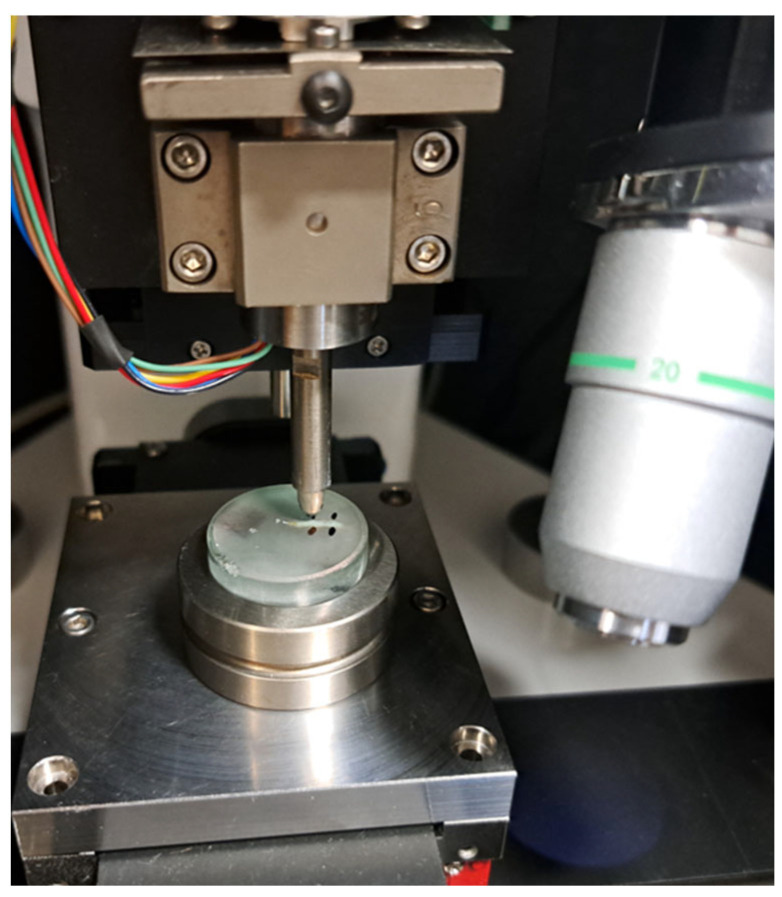
A representative image of a mounted rib specimen during nanoindentation, illustrating the experimental setup and sample positioning.

Subsequently, the dehydrated bones were embedded in Buehler two-component (epoxy cure 2) resin using cylindrical molds to facilitate controlled orientation and handling during mechanical testing. Once the resin had cured, samples were progressively ground and polished using abrasive papers (from p500 to p2000) of increasing fineness to achieve a smooth, flat surface suitable for the nanoindentation measurements. Finally, the polished samples were securely mounted onto a metal holder and subjected to both static and dynamic nanoindentation testing to determine mechanical parameters, including hardness, modulus of elasticity, and frequency-dependent viscoelastic properties (see Figure 1).

### 2.2. Nanoindentation Protocol

**Figure 2:**
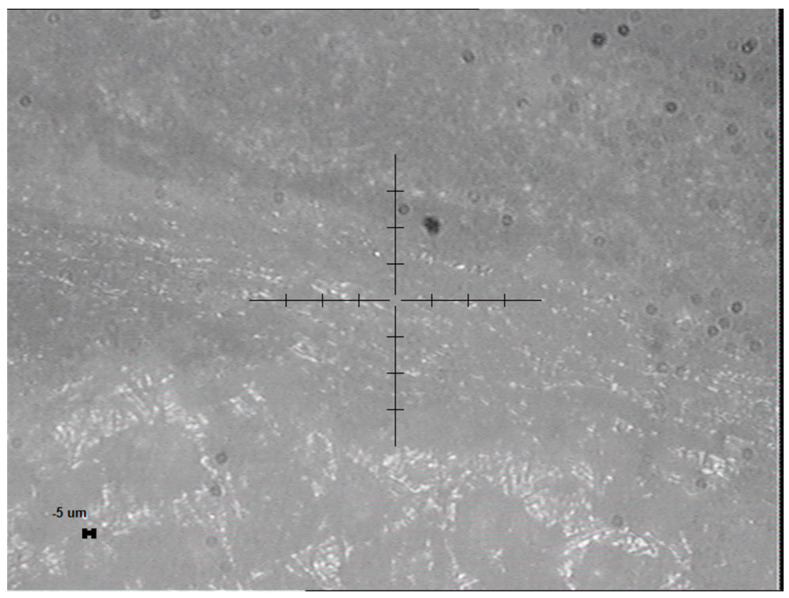
A representative optical microscope image of the rat rib bone sample nanoindentation site, captured using the built-in microscope of the Semilab IND-1500, showing the nanoindentation test site defined as the cortical bone between the embedding resin and the inner diploe (spongy bone) prior to nanoindentation testing.

Nanoindentation testing of the prepared rib bone samples was conducted using the Semilab IND-1500 nanoindenter (Semilab, Budapest, Hungary) equipped with a fresh Berkovich diamond tip (tip radius ~50 nm) also provided by Semilab ( $E_{ind}=1050\;GPa,\;\nu_{ind}=0.17$). The new area function of the tip was calibrated on standard fused silica, the compliance of the measurement device was calibrated and set as 0.258 nm/mN. The tests were performed under a controlled laboratory environment at 22 °C. In this study, the region of interest was the cortical bone; therefore, the indentation measurements were performed at the boundary of the cortical rib bones and embedding resin. The outer boundary of the rib near the resin interface was identified and targeted via the integrated optical microscope of the nanoindenter tool, see Figure 2. A load-controlled static indentation protocol was applied according to the ISO 14577 standard [23]. The $F_{max}$, maximum loading force was set to 10 mN with an initial contact of 15 µN followed by a 1 s hold at peak. The fitting for the modulus calculation was based on the upper 70% of the unloading curves, according to the standard procedure. On each sample, 20 static indentations were performed with a 20 µm spacing between the imprints to prevent interference from residual stress fields. For each indent, the load–displacement curves were recorded, and hardness (H) and the elastic modulus of the sample (E) were calculated using the Oliver–Pharr method based on the unloading curve [24,25,26] as follows:

(1) $$H=\frac{F_{max}}{A(h_{contact})}\;,$$

(2) $$E_{comb}=S\frac{\sqrt{\pi }}{2\sqrt{A_{contact}}},$$

where $A(h_{contact})$ is the contacted area and S is the contact stiffness. The contacted area was calculated from the $h_{contact}$ penetration depth and the geometry of the indenter tip. In our case, the $E_{indenter}\gg E_{sample}$ for all measurements. For the calculations, the Poisson’s ratio was taken as $\nu_{sample}=$ 0.3. The modulus of the sample can be calculated from the measured combined modulus as follows:

(3) $$\frac{1}{E_{comb}}=\frac{1-\nu_{sample}^{2}}{E_{sample}}+\frac{1-\nu_{indenter}^{2}}{E_{indenter}}.$$

Dynamic nanoindentation testing was performed to assess frequency-dependent viscoelastic properties, following the multi-frequency method described by Fisher-Cripps [5,21]. The dynamical response of the instrument device was calibrated using a reference spring to establish the TF transfer function for the equalization analysis, as follows:

(4) $$TF\;=\frac{TF_{mes}}{TF_{ref}}\;.$$

The dynamic indentations were also performed at the cortical bone region, aimed with the built-in optical microscope, see Figure 2. After a 15 µN approach on the sample surface, a fixed 10 mN load was applied, modulated by a pseudo-random force signal comprising 256 harmonics to induce oscillatory motion over 10 s at a 30% amplitude. The computer-generated command to the piezo of the instrument was used to apply the loading force, which was measured alongside the indentation depth via the independent LVDT voltage signals. On each sample, five dynamic loading tests were performed and averaged to enhance data reliability. Fourier analysis was employed to deconvolute the displacement and force signals into frequency-dependent quantities. The storage modulus (*E*′), loss modulus (*E*″), viscosity (*η*), and loss factor (tan *δ*) were evaluated based on the KelvinVoigt model. The complex modulus can be written in the following form:

(5) $$E^{*}=E\prime +iE^{\prime \prime }\;,$$

which, in case of the Kelvin–Voigt model, also satisfies the following:

(6) $$E^{*}=k-m\omega^{2}+i\omega \lambda \;,$$

where k is the contact stiffness, m is the effective indenter mass, λ is the damping coefficient (related to the *η* viscosity), and ω is the angular frequency (ω=2πf). Practically, the measured and equalized transfer function can be used to calculate the complex modulus and viscosity of the material as follows:

(7) $$E\prime \left(\omega \right)=\frac{\sqrt{\pi }}{2\sqrt{A}}\frac{\left|F_{0}\right|}{\left|h_{0}\right|}\cos\varphi =\frac{\sqrt{\pi }}{2\sqrt{A}}TF_{re}\;,$$

(8) $$E^{\prime \prime }\left(\omega \right)=\frac{\sqrt[{}]{\pi }}{2\sqrt[{}]{A}}\frac{\left|F_{0}\right|}{\left|h_{0}\right|}\sin\varphi =\frac{\sqrt[{}]{\pi }}{2\sqrt[{}]{A}}TF_{im}\;,$$

where |F|/|h| is the transfer function, and φ is the phase difference between them. For the dynamic parameters, the combined (specimen and indenter) values are reported, as Equation (3) was not applied in this case. Consequently, the absolute moduli are somewhat higher, since the contribution of the diamond indenter is included. However, this does not affect the comparative trends between the experimental groups. The loss factor, a quantity that measures the relative contributions of the storage and loss modulus to the mechanical response of the material, can be defined as follows:

(9) $$\tan\delta =\frac{E^{\prime \prime }}{E\prime }\;.$$

As described in the literature, tan *δ* > 1 indicates a predominantly viscous/fluid-like behavior, and tan *δ* < 1 indicates a solid-like response.

### 2.3. Surface Morphology and Characterization

Scanning electron microscope (SEM) secondary electron images were taken solely for visualization purposes using a Hitachi S-4700 (Hitachi High-Technologies Corporation, Tokyo, Japan) field emission cathode scanning electron microscope (FESEM). The surfaces of the bone specimens were coated with a few nm thin gold layers to eliminate the surface charging during the exposure.

### 2.4. Statistical Evaluation

Statistical analyses were performed using IBM SPSS Statistics (Version 23.0; IBM Corp., Armonk, NY, USA). Hardness and modulus of elasticity data were analyzed using one-way ANOVA to compare mechanical properties between the experimental groups, followed by a Bonferroni or Games–Howell post hoc test depending on the variance differences. Homogeneity of variances was tested with the Levene test. In the case of the dynamic loading test, the frequency-dependent behavior between the groups was compared with an ANCOVA test with the frequency as a covariate. In the case of the ANCOVA test, the Sidak post hoc test was used for the pairwise comparisons. For the statistical evaluations, the significance level was set as the standard *p* < 0.05. The results are reported as mean ± standard error of the mean.

## 3. Results

### 3.1. Results of the Static Nanoindentation

**Figure 3:**
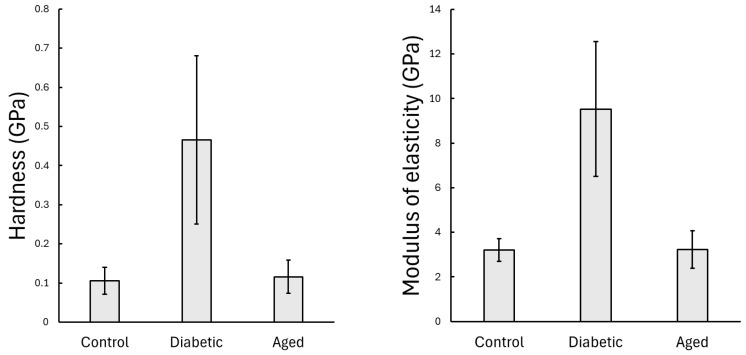
Results of the static nanoindentation, showing mean hardness and modulus of elasticity, and standard error as the error bar (*n* = 4 for young controls, *n* = 4 for diabetics, and *n* = 3 for old rats, 20 indentation sites per animal).

The hardness values of the rib bones were as follows: control group 0.106 ± 0.034 GPa, diabetic group 0.466 ± 0.215 GPa, and old-aged group 0.116 ± 0.042 GPa, see Figure 3. The one-way Welch ANOVA revealed a significant difference between the groups in the mean hardness (*p* < 0.001 *). The following Games–Howell post hoc test showed a statistically significant difference between the control and diabetic groups (*p* < 0.001 *), old-aged and diabetic (*p* < 0.001 *), but no significant difference between the control and old-aged groups (*p* = 0.696). The diabetic group showed a higher mean hardness value compared to the control and old-aged groups; however, the standard deviation ratio to the mean also increased, indicating a greater inhomogeneity in bone structure.

**Figure A1:**
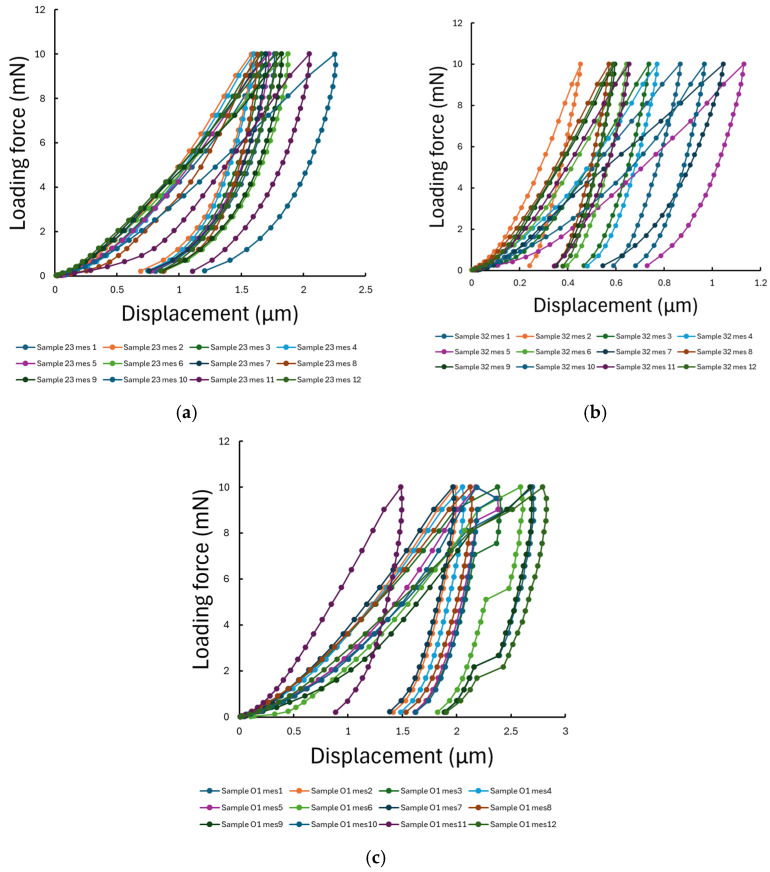
Nanoindentation results for rib bones. The first 12 load–displacement curves on the first animal of (**a**) control group, (**b**) diabetic group, (**c**) old group.

The modulus values for the rib bones were as follows: control group 3.21 ± 0.505 GPa, diabetic group 9.53 ± 3.03 GPa, and old-aged group 3.23 ± 0.84 GPa, see Figure 3. The Welch ANOVA also showed a statistically significant difference (*p* < 0.001 *) in the case of the mean modulus values between the groups. The following Games–Howell post hoc test revealed a similar tendency as the hardness—no significant difference in the case of the control and old-aged groups (*p* = 0.997), and a statistically significant difference between control and diabetic (*p* < 0.001 *), and old-aged and diabetic (*p* < 0.001 *). The higher modulus of elasticity in the diabetic group indicates greater bone stiffness, whereas the lower modulus values in the control and old-aged groups suggest a more flexible bone structure. Just as in the case of hardness, also in the case of modulus of elasticity, a higher standard deviation to mean ratio was observed in the case of diabetic bones compared to both control and old-aged groups, confirming the previously greater variability in mechanical properties statement in the case of diabetic rat rib bones. Representative load–displacement curves for all three groups are provided in the Appendix A section (see Figure A1). The contact stiffness and contact area used for the evaulation are provided in the Appendix B section.

The E/H modulus to hardness ratio is often used in nanoindentation studies to infer material properties like fracture toughness, as it reflects the balance between stiffness and resistance to plastic deformation. The 20.45 lower E/H ratio in the diabetic group indicates reduced fracture toughness, meaning a more fragile structure. The control group had a higher 30.34 ratio, suggesting a greater toughness, with the old-aged group showing an intermediate value of 27.83, showing that aging decreases the toughness of the bone structure as well.

**Figure 4:**
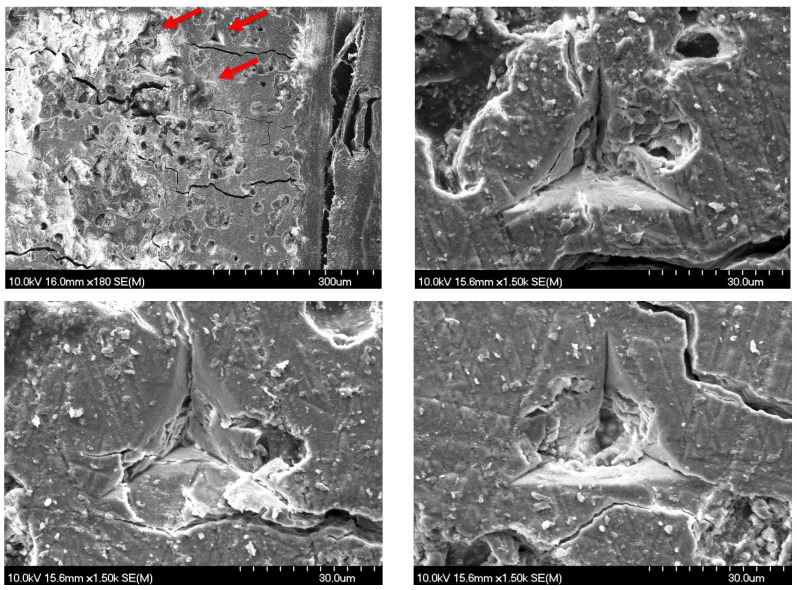
SEM images of diabetic rat rib bone after nanoindentation at different magnifications. The red arrows in the first image mark a few nanoindentation spots. Images were taken solely for visualization purposes.

SEM imaging of a sample of the diabetic group revealed the nanoindentation sites, showcasing the morphology of multiple indentations and also the surrounding bone microstructure, see Figure 4. The imprints of the indentations are displayed in triangular shapes due to the geometry of the Berkovich tip. The indentations were performed in the cortical part of the bone, and the images show a heterogeneous bone microstructure at this magnification.

### 3.2. Results of the Dynamic Nanoindentation

Dynamic nanoindentation was also performed on the samples to measure the loss factor (tanδ), storage modulus (E′), loss modulus (E″), and viscosity (η) until a frequency of 25.6 Hz with a high-resolution sampling (0.1 Hz step up for each 256 harmonics in 10 sec sampling time). Due to the mechanical resonance limitations of the measurement device, a frequency range of 0–4.5 Hz was used only from the data, and in this range, the device was calibrated by a spring reference transfer function for the evaluation. For the statistical evaluation, the frequency was taken as a covariate variable, and the groups were considered as the fixed factor.

**Figure 5:**
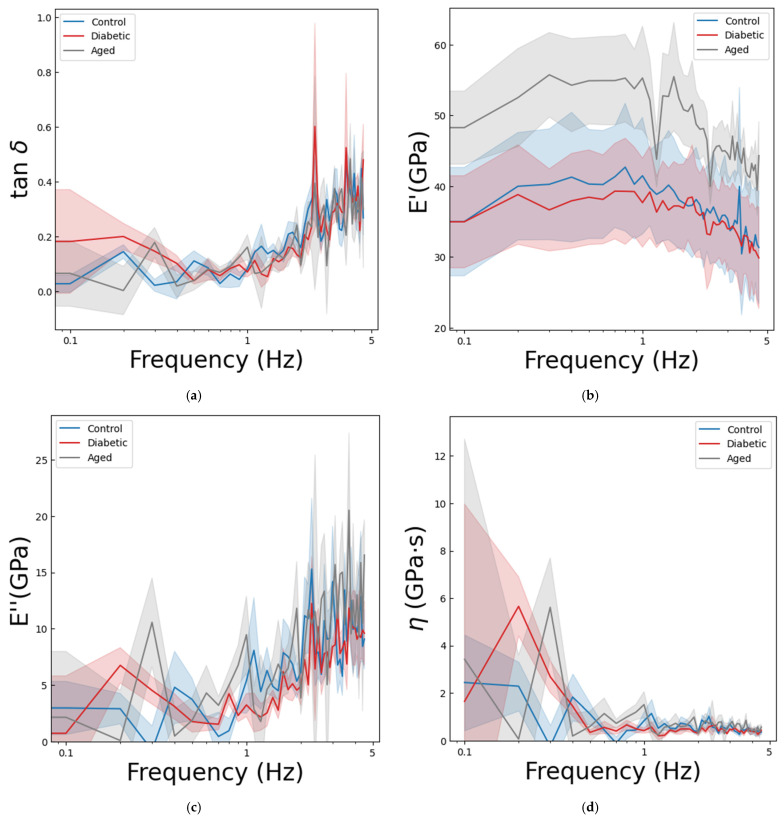
Frequency-dependent viscoelastic properties of rat ribs measured by dynamic nanoindentation: (**a**) mean tanδ (loss factor), (**b**) mean E′ (storage modulus), (**c**) mean E″ (loss modulus), (**d**) mean η (viscosity) as a function of frequency (log10 scale) for control (blue), old-aged (gray), and diabetic (red) groups. The colored bands represent the standard error of the mean (*n* = 4 for young controls, *n* = 4 for diabetics, and *n* = 3 for old rats, 20 indentation sites per animal).

The mean loss factor as a function of frequency is shown in Figure 5a. The ANCOVA statistical test, along with the Sidak post hoc tests, did not find a statistically significant difference in the mean loss factor between the groups (*p* = 0.525); only the frequency had a significant effect on the loss factors (*p* < 0.001 *), as can be seen also in the tendency in Figure 5a. An increase can be observed as a function of frequency, but the values are well below one, which indicates a solid-like response from the bone specimens.

The mean storage modulus values can be seen as a function of frequency until the 4.5 Hz cut-off in Figure 5b. The ANCOVA showed a statistically significant difference between the groups in the mean storage modulus over frequency (*p* < 0.001 *), and the frequency was also a significant covariate (*p* < 0.001 *). The pairwise Sidak post hoc tests revealed a significant difference between the control and old-aged groups (*p* < 0.001 *) and diabetic and old-aged groups (*p* < 0.001 *), whereas no statistically significant difference was observed between the control and diabetic groups (*p* = 0.502). In general, for the three groups, the mean storage modulus slightly increases at the very low frequency region and then decreases with a small slope until 4.5 Hz.

The mean loss modulus values can be seen as a function of frequency until 4.5 Hz in Figure 5c. The ANCOVA test showed a statistically significant difference between the groups (*p* < 0.001 *), and the frequency covariate variable was significant as well (*p* < 0.001 *). The pairwise Sidak comparisons showed only a significant difference in the case of diabetic and old-aged groups (*p* < 0.001 *). The difference between control and old-aged groups was not statistically significant (*p* = 0.056), but it suggests a potential trend toward a difference, while there was no statistically significant difference in the case of control and diabetic groups (*p* = 0.23). All three groups showed a linear tendency as a function of frequency.

The mean viscosity can be seen as a function of frequency until 4.5 Hz in Figure 5d. The ANCOVA statistical test, along with the Sidak post hoc tests, did not find a statistically significant difference in the mean loss factor between the groups (*p* = 0.699); the covariate frequency was significant in this case as well (*p* = 0.001 *). In general, the η viscosity rises highly in the low-frequency range, then decreases below 1 GPa∙s after 0.5 Hz, and oscillates around this value over frequency.

## 4. Discussion

Our study reveals distinct mechanical alterations in the rat rib cortical bone assessed by static and dynamic nanoindentation. To our knowledge, this is the first study to report both static and multifrequency dynamic nanoindentation measurements on rat rib bones, comparing diabetic, old, and control groups. Our static results showed that both the hardness and stiffness (modulus of elasticity) increased significantly in the presence of diabetes. In consequence, the ratio of the two decreased, resulting in a much lower fracture toughness in the case of the diabetic bones, making these bones more fragile in general. The dynamic results were focused on the frequency of the 0–4.5 Hz region, reflecting the physiological loading from respiration in rats. In this range, the frequency was a significant factor for all the mechanical parameters we examined. Dealing with the frequency as a covariant factor, the tan δ loss factor and η viscosity showed statistically no difference, indicating a similar solid-like behavior across all groups. However, both the E′ storage modulus and E″ loss modulus showed a significant difference between the groups, revealing that aging had a significant worsening effect on these quantities compared to the control or diabetic conditions, while diabetes did not have a significant effect on dynamic properties.

Casanova *et al*. investigated the micromechanical anisotropy of mice femur using nanoindentation [15]. Their polished, resin-embedded samples revealed that both hardness and elastic modulus were higher in the longitudinal direction compared to the transverse one, with hardness ranging from 0.38 to 0.82 GPa and modulus from 6.75 to 23.81 GPa. These values, obtained with standard surface preparation, confirm the applicability of nanoindentation for assessing elastic and plastic properties at the microscale. In our study, sample preparation involves an ethanol–xylene dehydration series, and the rib bones examined are generally less mineralized than the femur, which explains the lower absolute values but overall comparable trends.

Hu *et al.* combined nanoindentation and micro-CT analysis to characterize the trabecular region of L6 vertebrae in an ovariectomized rat model that mimics postmenopausal osteoporosis [27]. While estrogen deprivation did not significantly affect the nanomechanical properties (modulus of 24.6–25.3 GPa; hardness of 1.09–1.1 GPa), micro-CT revealed a dramatic change in the micro/nanoarchitecture of the bone structure. Their findings illustrate that structural degradation may precede measurable nanomechanical weakening, highlighting the complementarity of micro- and nanoscale analyses.

Sun *et al.* applied nanoindentation to femur bones of tail-suspended rats to model microgravity-induced bone loss [28]. The elastic modulus (9.2–10.7 GPa) and hardness (0.32–0.49 GPa) indicated reduced mechanical competence under unloading. The derived modulus-to-hardness ratio, a proxy for fracture toughness, changed significantly from 22.27 to 32.23. These values are consistent with our control (30.34) and diabetic (20.45) rib bone E/H ratios, supporting that nanoindentation can effectively detect matrix-level deterioration on bone under systemic or mechanical challenges.

Our results in rats align well with clinical bone fragility patterns in diabetes patients [29,30], emphasizing their translational relevance. The combination of higher hardness and markedly lower *E*/*H* ratio (a widely used nanoindentation surrogate of fracture toughness [31]) in diabetic rib bone reflects a stiffer but more brittle bone, consistent with the fragility paradox of normal or elevated bone mineral density accompanied by increased fracture risk [32]. This pattern is plausibly explained by the hyperglycemia-driven accumulation of AGEs in collagen, altering the collagen–mineral interactions, ultimately degrading bone quality [33]. Indeed, AGEs have already been proposed as biomarkers of fracture risk [11,34]. The wide variability observed in the mechanical properties of bone in the diabetic group highlights the true biological variation in bone mineralization related to diabetes. This can be a result of irregular mineralization (either related to intra-fibrillar mineral disruption by AGEs or more heterogeneous overall mineral distribution) [35,36], heterogeneous collagen degradation and modification of cross-links [37,38,39], and higher porosity in diabetic bones [40,41]. Indeed, the heterogeneous behavior of diabetic bone aligns with previous nanoindentation and compositional studies, supporting the increased scatter and spatial variability in bone mechanical and material properties found in our study [42].

The alteration in viscoelastic properties of aged bone, in contrast, occurred with unchanged static elastic modulus and hardness, which is consistent with multiscale findings that aging degrades collagen behavior [43]. Aging promotes accumulation of non-enzymatic collagen cross-links in bone collagen together with a decline in immature enzymatic cross-links, which can alter the dynamic response of the collagen–mineral–water matrix while leaving the small-strain elastic stiffness essentially unaffected [43,44]. In particular, the increased dynamic storage modulus likely reflects reinforced matrix stiffness due to an age-related accumulation of AGEs [12,45], whereas the higher loss modulus may reveal enhanced energy dissipation via porosity effects, molecular-scale viscoelastic mechanisms, or microstructural damping [45,46,47,48].

The combined results of the static and dynamic measurements suggest that in older patients with diabetes, the convergence of age-related alterations in collagen behavior and porosity-driven viscoelasticity with diabetes-induced increases in static stiffness may result in compounded degradation of bone quality, further elevating fracture risk.

While nanoindentation offers a precise means to assess nanoscale mechanical properties of bones, its direct applicability in clinical practice is constrained by the need for invasive sample preparation, small probing volumes, and specialized equipment. As such, translation to patient care is more feasible through higher-scale or non-invasive methods that can capture analogous information on bone quality. Techniques such as high-resolution peripheral quantitative computed tomography (HR-pQCT), magnetic resonance imaging, or vibrational spectroscopy (e.g., Raman or FTIR) may provide clinically relevant proxies for material properties measured at the nanoscale. Such combined evaluation of bone mass and quality could markedly improve fracture risk prediction in patients whose BMD is preserved yet whose bone quality is compromised, such as those with diabetes.

A few aspects of our work warrant consideration. First, while the sample sizes are relatively low (3–4 animals per group), the large number of indentation sites per sample (20 sites per animal) provided sufficient data for robust statistical analysis. The key between-group differences remained statistically significant despite the limited number of animals, indicating that the effects of diabetes and aging on bone mechanical behavior are strong and reproducible at the tissue level. Nevertheless, the inclusion of additional animals in future studies would improve statistical power, reduce inter-individual variability, and allow a more detailed assessment of biological heterogeneity. Second, while in vivo bones are hydrated, our measurements were performed on dehydrated samples. The effect of hydration on bone nanoindentation measurements has been well documented. Hydrated bone exhibits lower hardness and modulus values due to the plasticizing and lubricating effects of water on the collagen–mineral matrix [15,49]. Dehydration and embedding, as applied in the present study, are common procedures to ensure surface stability and reproducibility of nanoindentation tests [50,51]. Although absolute values of mechanical parameters are therefore expected to be higher in dried specimens, dehydration does not fundamentally alter the mineral phase or the collagen cross-link network that governs intergroup differences. Consequently, the relative trends between groups can still be considered biologically meaningful. This interpretation is consistent with previous studies showing that while drying stiffens the tissue by roughly 30–50%, the hierarchical pattern of stiffness variations within and between bones remains unchanged [50,52]. Moreover, hardness is typically more affected by dehydration than modulus, whereas viscoelastic and damping properties decrease due to the loss of bound water that mediates collagen-mineral sliding [53,54]. Therefore, although our measurements represent the dry-state response, the comparative differences observed between experimental groups are expected to reflect true biological alterations rather than artifacts of sample preparation.

## 5. Conclusions

This study demonstrates that diabetes significantly alters the nanomechanical properties of rat rib cortical bone. Our findings suggest that diabetes induces both structural and intrinsic material changes, distinct from age-related effects. The diabetic condition increases the stiffness and hardness of the bones, while decreasing the fracture toughness dramatically. In the physiological loading frequency region, the mechanical properties do not change significantly compared to control animals; however, old age had a stronger effect in this case on the storage and loss moduli.

## Data Availability

The original raw data contributions presented in this study are included in the article/Supplementary Material. Further inquiries can be directed to the corresponding author.

## Supplementary Materials

The following supporting information can be downloaded at: https://www.mdpi.com/article/10.3390/nano15201582/s1. The supplementary file contains the raw nanoindentation data used for the analyses presented in this study.

## Appendix A. Representative Load–Displacement Curves

Representative load–displacement curves for all three groups are provided in the Appendix (see Figure A1) to illustrate the nanoindentation behavior of the rib specimens. In case of control and old-aged samples the displacement at the maximum of 10 mN loading goes higher than 1 µm while in case of diabetic samples it can be seen this is below 1 µm and also the variability is higher.

## Appendix B. Supplementary Nanoindentation Parameters

For completeness, the mean contact stiffness (S) and contact area ($A_{c}$) from the static nanoindentation measurements are summarized. These values served as the basis for calculating the hardness and modulus of elasticity according to the Oliver–Pharr method. The contact stiffness mean value along with the standard error of the mean was 36.97 ± 3.93 mN/µm for the control group, 57.38 ± 7.14 mN/µm for the diabetic group, and 39.053 ± 4.9 mN/µm for the older people group. The mean contact areas were 100.79 ± 33.16 µm^2^ for the control group, 42 ± 15.54 µm^2^ for the diabetic group, and 115.6 ± 34.99 µm^2^ for the older people group.
