# Supplementary figures and images for: Nanomechanical Properties of Rib Bones in Diabetic vs. Healthy Rat Models
Source: Nanomaterials (Basel). 2025 Oct 17;15(20):1582. doi: 10.3390/nano15201582 (PMC12566810; doi:10.3390/nano15201582)

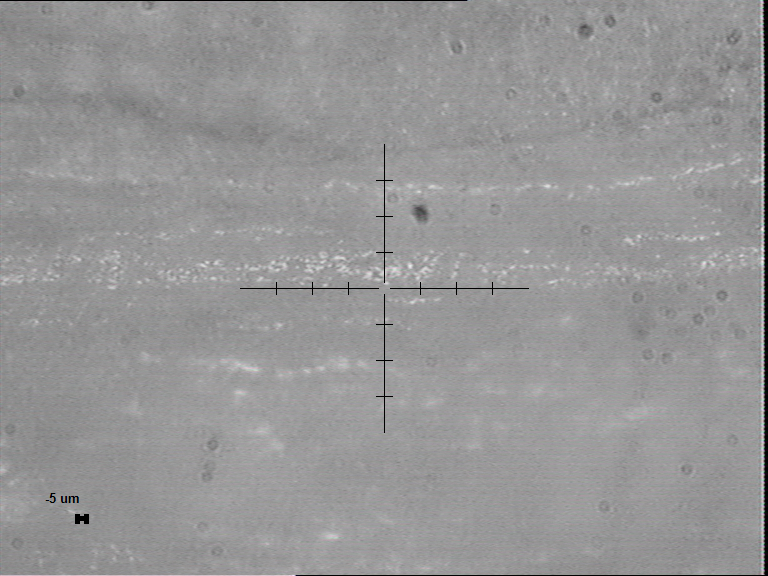

Supplement: Supplementary file 1 [file nanomaterials-15-01582-s001.zip › Nanoindentation data/raw static/sample 23/Image1.jpg]

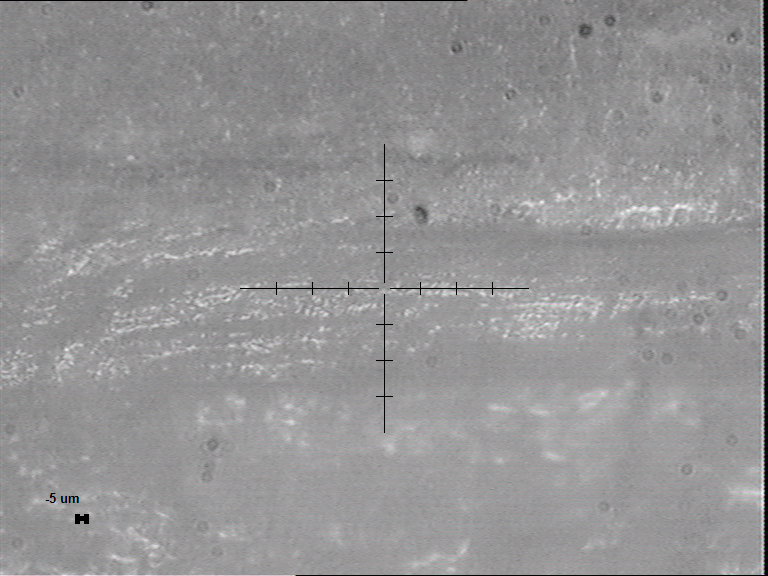

Supplement: Supplementary file 1 [file nanomaterials-15-01582-s001.zip › Nanoindentation data/raw static/sample 23/Image2.jpg]

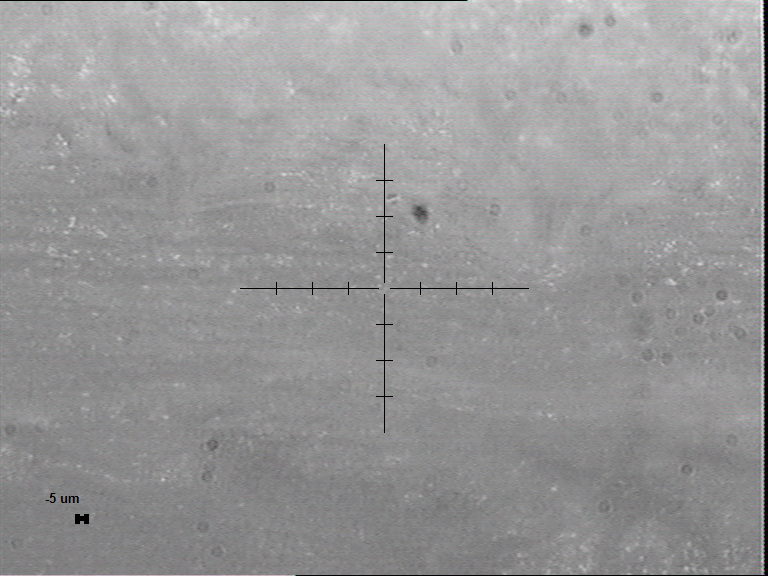

Supplement: Supplementary file 1 [file nanomaterials-15-01582-s001.zip › Nanoindentation data/raw static/sample 24/Image1.jpg]

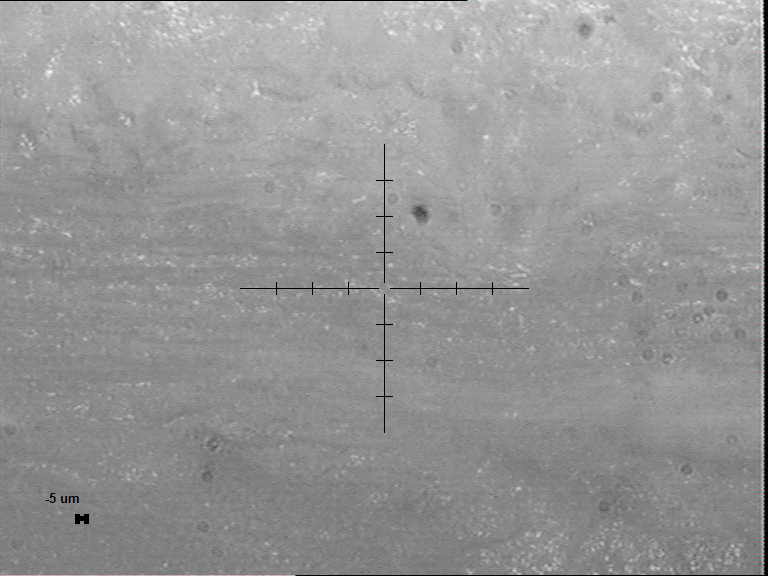

Supplement: Supplementary file 1 [file nanomaterials-15-01582-s001.zip › Nanoindentation data/raw static/sample 24/Image1post.jpg]

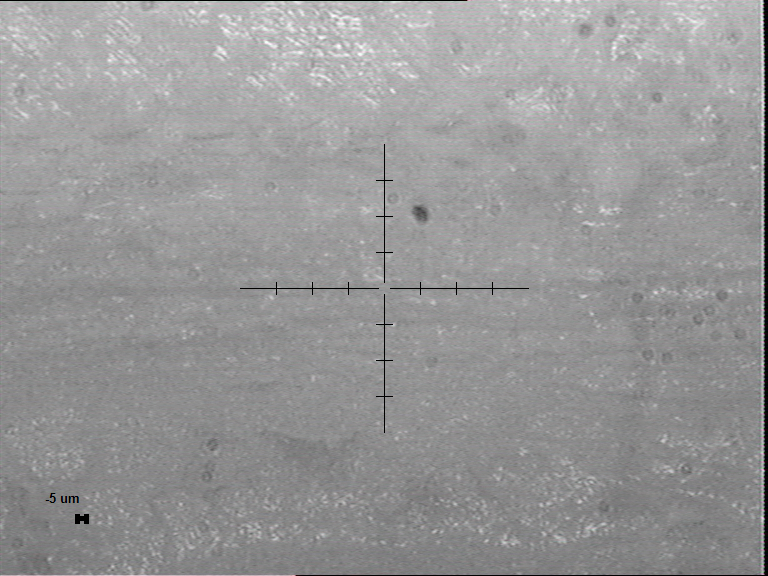

Supplement: Supplementary file 1 [file nanomaterials-15-01582-s001.zip › Nanoindentation data/raw static/sample 24/Image2.jpg]

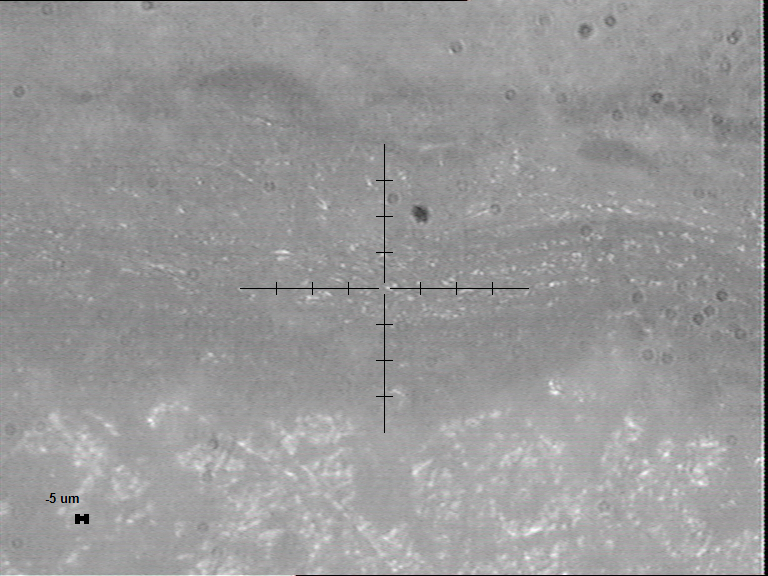

Supplement: Supplementary file 1 [file nanomaterials-15-01582-s001.zip › Nanoindentation data/raw static/sample 25/Image1.jpg]

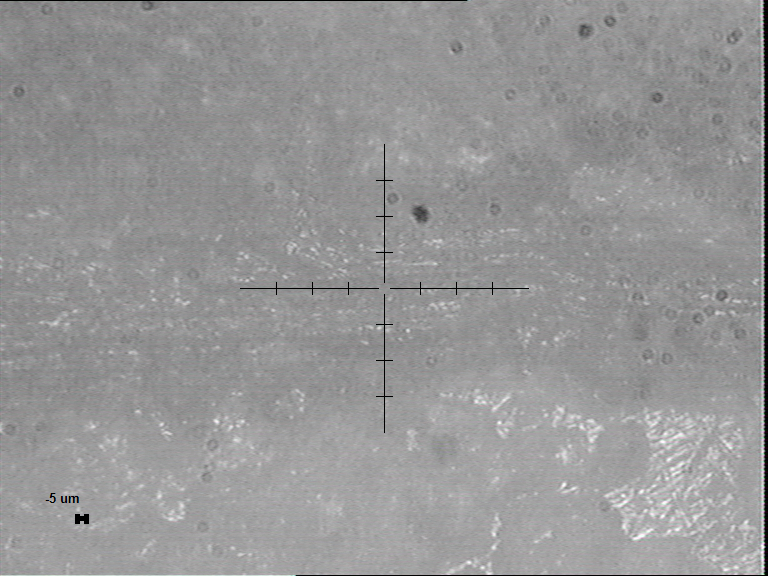

Supplement: Supplementary file 1 [file nanomaterials-15-01582-s001.zip › Nanoindentation data/raw static/sample 25/Image2.jpg]
